# Risk factors for testicular germ cell tumours by histological tumour type

**DOI:** 10.1038/sj.bjc.6690611

**Published:** 1999-08

**Authors:** C A C Coupland, C E D Chilvers, G Davey, M C Pike, R T D Oliver, D Forman

**Affiliations:** Division of Public Health Medicine and Epidemiology, School of Community Health Sciences, University of Nottingham Medical School, Nottingham, NG7 2UH, UK; ICRF Cancer Epidemiology Unit, Radcliffe Infirmary, Oxford, OX2 6HE, UK; Department of Preventive Medicine, University of Southern California School of Medicine, Los Angeles, California, 90033-0800, USA; Department of Medical Oncology, The Royal London Hospital, London, ECIA 7BE, UK

**Keywords:** case-control study, histology, risk factors, seminoma, testicular cancer

## Abstract

There are two main histological groups of testicular germ cell tumours, which may have different risk factors. Some authors have analysed potential risk factors by histological group but few consistent differences have been identified. In this paper we examine risk factors for pure seminoma and other tumours using data from the United Kingdom case control study of testicular cancer. Seven hundred and ninety-four cases were included in the study, each with a matched control; 400 cases had pure seminoma tumours, and 394 had other testicular tumours. The risk of seminoma associated with undescended testis was slightly higher than that for other tumours (odds ratio of 5.3 compared with 3.0). When split at the median age at diagnosis, this difference was greater in men aged 32 and over (odds ratio of 11.9 compared with 5.1) than in the younger men (3.0 compared with 2.5). Risks associated with testicular or groin injuries were higher in the non-seminoma group, as was the risk for a history of sexually transmitted disease. The protective effect of a late puberty was more marked for tumours of other histologies. Some differences were also detected for participation in sports. Whilst some of the differences detected may have arisen by chance, the stronger association between undescended testis and pure seminoma has been identified by a number of other studies and may reflect a genuine difference in aetiology. © 1999 Cancer Research Campaign


British Journal of Cancer (1999) **80**(11), 1859–1863
© 1999 Cancer Research Campaign
Article no. bjoc.1999.0611

### Risk factors for testicular germ cell tumours by histological tumour type

**CAC Coupland[1], CED Chilvers[1], G Davey[2], MC Pike[3], RTD Oliver[4] and D Forman[2]† on behalf of the United Kingdom Testicular Cancer Study Group***

[1]Division of Public Health Medicine and Epidemiology, School of Community Health Sciences, University of Nottingham Medical School, Nottingham NG7 2UH, UK; [2]ICRF Cancer Epidemiology Unit, Radcliffe Infirmary, Oxford OX2 6HE, UK; [3]Department of Preventive Medicine, University of Southern California School of Medicine, Los Angeles, California 90033-0800, USA; [4]Department of Medical Oncology, The Royal London Hospital, London ECIA 7BE, UK

**Summary** There are two main histological groups of testicular germ cell tumours, which may have different risk factors. Some authors have analysed potential risk factors by histological group but few consistent differences have been identified. In this paper we examine risk factors for pure seminoma and other tumours using data from the United Kingdom case control study of testicular cancer. Seven hundred and ninety-four cases were included in the study, each with a matched control; 400 cases had pure seminoma tumours, and 394 had other testicular tumours. The risk of seminoma associated with undescended testis was slightly higher than that for other tumours (odds ratio of 5.3 compared with 3.0). When split at the median age at diagnosis, this difference was greater in men aged 32 and over (odds ratio of 11.9 compared with 5.1) than in the younger men (3.0 compared with 2.5). Risks associated with testicular or groin injuries were higher in the non-seminoma group, as was the risk for a history of sexually transmitted disease. The protective effect of a late puberty was more marked for tumours of other histologies. Some differences were also detected for participation in sports. Whilst some of the differences detected may have arisen by chance, the stronger association between undescended testis and pure seminoma has been identified by a number of other studies and may reflect a genuine difference in aetiology.

**Keywords:** case-control study; histology; risk factors; seminoma; testicular cancer

The United Kingdom Testicular Cancer Case-control Study is one of the largest studies of the aetiology of testicular germ cell tumours carried out to date. The main associations with testicular tumour risk previously reported from this study were for a history of undescended testis or inguinal hernia, an early age at puberty and lack of exercise (UK Testicular Cancer Study Group, 1994*a*) in addition to testicular trauma and a history of sexually transmitted disease (UK Testicular Cancer Study Group, 1994*b*). These results combined the two histological groups of testicular tumours, namely pure seminoma and other tumours. The different age-profiles at diagnosis for these two groups as well as some findings from other studies suggest possible differences in the aetiology of these tumour types.

A preliminary analysis of differences by histological tumour type using data from the United Kingdom Testicular Cancer Case-control Study suggested some factors where risks differed significantly by tumour type (Coupland et al, 1998). These findings were derived using matched analyses whereby each histological group of tumour cases was compared with its own matched set of controls. In these analyses we found significant differences in the distribution of certain variables between the two control groups, which made any differences between the histological groups hard to interpret. Further analysis was therefore necessary, and the final results of this analysis are reported here.

### MATERIALS AND METHODS

The study was carried out in nine health regions within the UK. Full details of the study design have been previously published (UK Testicular Cancer Study Group, 1994*a*). In brief, a geographical area was defined in each region. All men diagnosed with a testicular germ cell tumour between 1 January 1984 and 30 September 1986 (with some regional variation), aged 15–49 years at diagnosis and resident in the study areas were included. The main sources for identification of cases were major treatment centres and regional cancer registries. The study was restricted to white men with no previous malignancy or severe mental disability.

One control was interviewed for each case, selected from the list of the general practitioner (GP) with whom the case was registered and matched by date of birth to within 1 year. Each matched pair was seen by the same interviewer.

The interview included questions on medical history, sexual development and marital history, personal lifestyle including participation in sports and exercise, and occupational history. Most questions referred to events happening up to 1 year before the diagnosis of the case or the equivalent date for their age-matched control. The reference age was defined as the age of the case and

*Received 22 October 1998*
*Revised 22 February 1999*
*Accepted 23 February 1999*

*Correspondence to:* CAC Coupland

*Remaining members are given at the end of the article
†Current address: Centre for Cancer Research, University of Leeds, Cookridge Hospital, Leeds LS16 6QB, UK..

matched control 1 year before the diagnosis date, or the equivalent date for the control.

After the interview and with the subjects' consent, further data on medical history were abstracted from their general practitioner notes, permission was also sought to send postal questionnaires to the subjects' mothers to collect information on their sons' health as a child. Both the general practitioner notes and the mothers' questionnaires were used to confirm a history of undescended testis, inguinal hernia and testicular trauma.

Details of the tumours of the cases were abstracted from their hospital notes and copies of their pathology reports were obtained. These reports were reviewed centrally to determine the histological type of tumour. Tumours were classified as 'pure seminoma' or 'other histological type', the latter group including tumours with mixed histologies.

The preliminary analyses by histological group used conditional logistic regression for matched studies to estimate odds ratios for the two histological groups of cases and their matched controls separately. There were, however, considerable differences in the distribution of certain variables between the group of controls matched to the pure seminoma cases and the group matched to cases with other histological tumours (Coupland et al, 1998), the prevalence of undescended testis, for example, was higher in controls matched to cases of other histologies (3.6%) compared with controls matched to seminoma cases (0.8%), a difference which remained statistically significant after adjustment for age group, study area and social class ($P$ = 0.03). There were also differences in the distribution of testicular trauma-related variables between the two control groups. In order that differences between the histological groups could be estimated without being unduly influenced by differences between the control groups, the analyses reported here used unconditional logistic regression, which broke the matching and compared each histological group of cases with all the controls. The odds ratios (OR) and 95% confidence intervals (CI) calculated were adjusted for age (at diagnosis or equivalent, in 5-year age bands), study area and social class (categorized into seven groups). Adjustment was also carried out where stated for a history of undescended testis and inguinal hernia (before age 15). Significance tests (tests of heterogeneity) were also calculated based on a direct comparison of the two groups of cases, adjusted for the variables listed above in order to identify risks which differed significantly by tumour type using the likelihood ratio test in an unconditional logistic regression. The significance values reported are two-sided. Analyses were performed only for variables either previously found to be significant for all tumours combined (UK Testicular Cancer Study Group, 1994*a*, 1994*b*), or identified by other authors to have different associations by histological group. This strategy was chosen to reduce the chance of detecting false-positive associations.

### RESULTS

A total of 863 eligible cases were identified and 794 of these (92.0%) were interviewed, as were 609 (76.7%) of the 794 first-selected controls and 185 replacement controls (UK Testicular Cancer Study Group, 1994*a*). There were 400 cases with tumours described as pure seminoma and 394 with tumours of other germ cell histological types (including 77 of mixed histology). The median age at diagnosis was 35 for pure seminoma, 28 for non-seminoma and 32 for all tumours.

As previously reported (UK Testicular Cancer Study Group, 1994*a*) the odds ratio associated with a history of undescended testis for all testicular cancer cases was 3.82 (95% CI = 2.24–6.52). Using an unmatched analysis, the adjusted OR for pure seminoma associated with undescended testis of 5.30 (95% CI = 2.89–9.73), did not differ significantly from the OR of 3.00 (95% CI = 1.59–5.63) for other histologies (Table 1). The adjusted OR associated with undescended testis in the 77 mixed tumour cases was 5.15 (95% CI = 2.08–12.75), and in the remaining non-seminoma cases it was 2.46 (95% CI = 1.24–4.91).

In cases with a history of undescended testis, there was a significantly higher proportion of bilateral undescended testis in pure seminoma cases (39.5%) than in the other cases (14.8%), among which 12.5% of the mixed tumour cases had bilateral undescended testis. In addition, in cases with unilateral undescended testis, a higher proportion of pure seminoma cases had a late corrected (15 or older) or uncorrected testis (47.8% compared with 26.1% in all other tumours) although this difference was not statistically significant. The overall risk of testicular cancer associated with a history of undescended testis when split at the median age at diagnosis (32 years) was higher in the older (OR = 8.25) than the younger men (OR = 2.46) (UK Testicular Cancer Study Group, 1994*a*). In men aged younger than 32 the OR associated with undescended testis were 3.03 for pure seminoma tumours and 2.46 for all other tumours, whereas in the older men they were 11.94 and 5.10 respectively, the latter difference being of borderline

**Table 1** Odds ratios for undescended testis by age-group and histological tumour type

| Undescended testis | Pure seminoma Cases *n* | (%) | Odds ratio[a] (95% CI) | Other histologies Cases n | (%) | Odds ratio[a] (95% CI) | All controls *n* | (%) | Significance test[a,b] |
|---|---|---|---|---|---|---|---|---|---|
| All men | | | | | | | | | |
| No | 362 | (90.5) | 1.00 | 367 | (93.1) | 1.00 | 777 | (97.9) | $P$ = 0.16 |
| Yes | 38 | (9.5) | 5.30 (2.89–9.73) | 27 | (6.9) | 3.00 (1.59–5.63) | 17 | (2.1) | |
| Men aged <32 | | | | | | | | | |
| No | 121 | (91.0) | 1.00 | 242 | (92.4) | 1.00 | 382 | (96.7) | $P$ = 0.76 |
| Yes | 12 | (9.0) | 3.03 (1.30–7.06) | 20 | (7.6) | 2.46 (1.19–5.10) | 13 | (3.3) | |
| Men aged 32+ | | | | | | | | | |
| No | 241 | (90.3) | 1.00 | 125 | (94.7) | 1.00 | 395 | (99.0) | $P$ = 0.056 |
| Yes | 26 | (9.7) | 11.94 (4.04–35.28) | 7 | (5.3) | 5.10 (1.43–18.25) | 4 | (1.0) | |

[a]Odds ratios, 95% confidence intervals (CI) and significance tests adjusted for age-group, study area and social class. [b]Significance test calculated using likelihood ratio test in comparison of two groups of cases.

© 1999 Cancer Research Campaign

**Table 2** Odds ratios for inguinal hernia, testicular trauma and sexually transmitted disease by histological tumour type

| Variable | Response | Pure seminoma Cases *n* | (%) | Odds ratio[a] (95% CI) | Other histologies Cases *n* | (%) | Odds ratio[a] (95% CI) | All controls *n* | (%) | Significance test[a,b] |
|---|---|---|---|---|---|---|---|---|---|---|
| Inguinal hernia[c] | No | 340 | (93.9) | 1.00 | 346 | (94.3) | 1.00 | 752 | (96.8) | $P$ = 0.59 |
| | Yes | 22 | (6.1) | 1.60 (0.88–2.93) | 21 | (5.7) | 2.39 (1.28–4.46) | 25 | (3.2) | |
| Inguinal hernia | <15 years | 17 | (4.7) | 3.12 (1.42–6.88) | 12 | (3.3) | 2.49 (1.06–5.88) | 11 | (1.4) | $P$ = 0.020[d] |
| Age at diagnosis[c] | 15+ years | 5 | (1.4) | 0.56 (0.20–1.60) | 9 | (2.5) | 2.28 (0.93–5.56) | 14 | (1.8) | |
| Testis or groin injury for which | | | | | | | | | | |
| (a) Took at least 1 day | No | 388 | (97.0) | 1.00 | 371 | (94.2) | 1.00 | 775 | (97.6) | $P$ = 0.046 |
| off work or school | Yes | 12 | (3.0) | 1.39 (0.66–2.94) | 23 | (5.8) | 2.66 (1.41–5.04) | 19 | (2.4) | |
| (b) Consulted GP | No | 390 | (97.5) | 1.00 | 375 | (95.2) | 1.00 | 770 | (97.0) | $P$ = 0.023 |
| | Yes | 10 | (2.5) | 0.89 (0.41–1.93) | 19 | (4.8) | 1.67 (0.89–3.14) | 24 | (3.0) | |
| (c) Went to hospital | No | 392 | (98.0) | 1.00 | 377 | (95.7) | 1.00 | 779 | (98.1) | $P$ = 0.013 |
| | Yes | 8 | (2.0) | 1.19 (0.49–2.89) | 17 | (4.3) | 2.45 (1.18–5.08) | 15 | (1.9) | |
| Ever had any sexually | No | 367 | (92.2) | 1.00 | 348 | (88.5) | 1.00 | 755 | (95.1) | $P$ = 0.018 |
| transmitted disease | Yes | 31 | (7.8) | 1.55 (0.93–2.59) | 45 | (11.5) | 2.93 (1.84–4.67) | 39 | (4.9) | |

[a]Odds ratios, 95% confidence intervals (CI) and significance tests adjusted for age-group, study area and social class and undescended testis or inguinal hernia diagnosed < 15 years (but see note c). [b]Significance test calculated using likelihood ratio test in comparison of two groups of cases. [c]Adjusted for age-group, study area and social class, all men with undescended testis excluded from analysis. [d]Significance test using 3 categories of response: no hernia, diagnosed at <15 years, and diagnosed at 15+ years.

**Table 3** Odds ratios for sexual development and sports participation by histological tumour type

| Variable | Response | Pure seminoma Cases *n* | (%) | Odds ratio[a] (95% CI) | Other histologies Cases *n* | (%) | Odds ratio[a] (95% CI) | All controls *n* | (%) | Significance test[a,b] |
|---|---|---|---|---|---|---|---|---|---|---|
| Age at first | <13 | 46 | (13.7) | 1.00 | 53 | (16.8) | 1.00 | 85 | (13.5) | $P$ = 0.051[c] |
| nocturnal | 13 | 56 | (16.7) | 1.07 (0.64–1.78) | 62 | (19.6) | 0.98 (0.61–1.59) | 103 | (16.4) | |
| emissions | 14 | 82 | (24.4) | 1.24 (0.77–2.00) | 70 | (22.2) | 0.80 (0.50–1.28) | 130 | (20.7) | |
| (years) | 15 | 44 | (13.1) | 0.90 (0.53–1.52) | 38 | (12.0) | 0.62 (0.37–1.04) | 100 | (15.9) | |
| | 16+ or never | 108 | (32.1) | 1.04 (0.66–1.64) | 93 | (29.4) | 0.67 (0.44–1.05) | 210 | (33.4) | |
| Age voice | <13 | 45 | (16.4) | 1.00 | 54 | (19.5) | 1.00 | 80 | (14.5) | $P$ = 0.071[c] |
| broke (years) | 13 | 73 | (26.5) | 0.93 (0.58–1.51) | 89 | (32.1) | 1.00 (0.64–1.58) | 137 | (24.9) | |
| | 14 | 97 | (35.3) | 1.02 (0.64–1.61) | 78 | (28.2) | 0.66 (0.42–1.03) | 177 | (32.2) | |
| | 15 | 37 | (13.5) | 0.80 (0.46–1.40) | 38 | (13.7) | 0.68 (0.40–1.15) | 85 | (15.5) | |
| | 16+ or not yet | 23 | (8.4) | 0.61 (0.33–1.14) | 18 | (6.5) | 0.34 (0.18–0.66) | 71 | (12.9) | |
| Participation in sports | | | | | | | | | | |
| At age 16: | Athletics | 65 | (16.3) | 0.74 (0.52–1.04) | 89 | (22.8) | 1.20 (0.88–1.63) | 155 | (19.7) | $P$ = 0.081 |
| | Contact sports | 241 | (60.4) | 1.02 (0.79–1.33) | 244 | (62.6) | 1.03 (0.79–1.33) | 482 | (61.3) | $P$ = 0.88 |
| | Racquet sports | 60 | (15.0) | 0.96 (0.67–1.38) | 77 | (19.7) | 1.07 (0.76–1.49) | 132 | (16.8) | $P$ = 0.70 |
| At age 20: | Athletics | 30 | (7.7) | 0.74 (0.46–1.19) | 37 | (10.9) | 1.10 (0.71–1.70) | 71 | (9.7) | $P$ = 0.11 |
| | Contact sports | 133 | (33.9) | 0.71 (0.54–0.93) | 134 | (39.5) | 0.91 (0.69–1.19) | 306 | (41.9) | $P$ = 0.033 |
| | Racquet sports | 56 | (14.3) | 0.87 (0.60–1.25) | 72 | (21.2) | 1.27 (0.90–1.80) | 123 | (16.8) | $P$ = 0.052 |
| At reference age: | Athletics | 25 | (6.4) | 0.54 (0.33–0.86) | 49 | (12.5) | 0.95 (0.65–1.39) | 101 | (12.9) | $P$ = 0.091 |
| | Contact sports | 41 | (10.4) | 0.52 (0.35–0.78) | 83 | (21.2) | 0.87 (0.64–1.19) | 158 | (20.1) | $P$ = 0.048 |
| | Racquet sports | 85 | (21.6) | 1.08 (0.78–1.50) | 103 | (26.3) | 1.53 (1.12–2.08) | 160 | (20.4) | $P$ = 0.052 |

[a]Odds ratios, 95% confidence intervals (CI) and significance tests adjusted for age group, study area, social class and undescended testis or inguinal hernia diagnosed < 15 years. [b]Significance test calculated using likelihood ratio test in comparison of two groups of cases. [c]Trend test across categories as shown in Table.

statistical significance (Table 1). For the mixed tumours the OR were 2.98 and 13.28 for younger and older men respectively.

The OR for inguinal hernia were similar for pure seminoma and other tumours (Table 2); however, when subdivided by age at diagnosis of hernia the risk associated with hernias diagnosed after the age of 15 was higher for non-seminoma tumours than for pure seminoma.

The OR for the only other medical factors which differed significantly by tumour type are also shown in Table 2. The risks associated with testicular trauma were greater for histologies other than pure seminoma, this being so for injuries requiring at least 1 day's absence from work or school and those requiring a general practitioner consultation or hospitalization (all $P < 0.05$). There was an increased risk of non-seminoma (OR = 2.93) compared with pure seminoma (OR = 1.55) associated with ever having had a sexually transmitted disease ($P = 0.018$).

The OR for the sexual development and lifestyle variables which differed by histological group are shown in Table 3. The protective effect of later puberty identified for all testicular tumours (UK Testicular Cancer Study Group, 1994*a*) was marginally stronger for non-seminoma tumours for the age at first nocturnal emissions and the age at which the voice broke. Participation in contact sports at age 20 and at the reference age was more protective for pure seminoma tumours than for non-seminoma tumours and there were differences of borderline significance for participation in racquet sports and athletics at some ages. There were, however, no significant differences in risk between the two histological groups associated with time spent participating in exercise per week or spent sitting down each day (data not shown) which were significant overall both at age 20 and at the reference age (UK Testicular Cancer Study Group, 1994*a*).

There were no significant differences in risk by histological group according to employment for 5 or more years in any of 16 standard occupational groups.

### DISCUSSION

Most studies of testicular tumours have considered tumours of all histological types as a single group in their analyses, as we did in our previous reports on the United Kingdom Testicular Cancer Case-control Study (UK Testicular Cancer Study Group, 1994*a*, 1994*b*). There are, however, some indications that the risk factor profiles may differ by histological group since several authors have found some differing risk factors for the histological groups, although few used formal statistical tests to identify significant histological differences. It is unlikely, however, that differences in aetiology would be large since both groups show similar trends in incidence (Møller, 1993). In our analyses, as in other studies, a number of variables were explored, so there is an increased risk of identifying spurious differences by chance alone. Therefore, in the interpretation of our results we attach more importance to findings consistent with those from other studies.

We found a stronger, but not statistically significant, association between a history of undescended testis and pure seminoma than non-seminoma tumours, although there was a larger difference in risk, of borderline statistical significance, in an analysis restricted to older men (OR of 11.9 and 5.1). Among the authors who have examined risks separately, Morrison (1976), Stone et al (1991) and Prener et al (1996) found a significantly stronger association with seminoma, Morrison (1976) for example reported OR of 15.6 for pure seminoma compared with 5.3 for non-seminoma, and Prener et al (1996) reported OR of 7.3 and 3.6 respectively. Henderson et al (1979), Moss et al (1986), Swerdlow et al (1987), Strader et al (1988), Haughey et al (1989), Gallagher et al (1995) and Møller et al (1996) reported similar risks associated with undescended testis in the two histological groups, although where separate OR were presented, they were consistently higher for seminomas than other tumours (Moss et al, 1986; Swerdlow et al, 1987; Strader et al, 1988; Møller et al, 1996). In a case-control study only of pure seminoma tumours (Coldman et al, 1982) the OR associated with undescended testis was 17.1. These studies used various definitions of undescended testis including self-report and physician assessment. Our definition required either evidence of orchidopexy or lack of descent at the reference age and so was not susceptible to recall bias. The overall consistency in the pattern of risk from all these reports for undescended testis implies a genuine difference with a higher risk for pure seminoma. Our finding of an excess of bilateral undescended tests in seminoma cases adds further support to this being a real effect. It is of interest that the risk associated with undescended testis in men with mixed tumours was similar to that for pure seminoma, although conventionally these tumours are grouped with non-seminoma tumours. This would suggest considering this group separately where numbers are sufficient, as proposed by Oliver et al (1995). Two other studies (Swerdlow et al, 1987; Stone et al, 1991) have found, as we did a tendency for late corrected or uncorrected undescended testis to predispose to pure seminoma, the consistency of these findings, albeit in a rather small subgroup, again suggests a real effect.

The risks associated with inguinal hernia were similar in the two histological groups overall in our analyses. An examination according to the age at diagnosis of the hernia, however, revealed differing patterns of risk. Risks were raised, and of similar magnitude, for hernias diagnosed before the age of 15, whilst for hernias diagnosed later than this the risk was increased for non-seminoma tumours (OR = 2.3), but reduced for pure seminomas (OR = 0.6). In the study by Swerdlow et al (1987) an increased odds ratio for seminoma (OR = 3.8) was associated with childhood herniorrhaphy before age 15, whereas no association was found with non-seminoma tumours. Similarly, Prener et al (1996) reported an OR for hernias diagnosed before 15 years of age of 2.3 for pure seminoma compared with 1.2 for other tumours. Morrison (1976), Haughey et al (1989) and Gallagher et al (1995) found no difference in risk. There is no clear and consistent pattern in these results, and although there is an indication that hernias diagnosed in childhood may be more strongly associated with seminoma any true difference in risk is unlikely to be large.

For three variables examining the effect of testicular trauma we found risks were significantly increased for non-seminoma tumours, compared with pure seminoma tumours. This remained so when analyses were restricted to men either above or below the median age. In order to reduce recall bias for testicular trauma, mother's questionnaires and GP notes were used to verify these reports. Only two other studies to our knowledge have investigated the role of trauma by histological group, the study by Stone et al (1991) found, like us, increased levels of trauma in non-seminoma cases, whereas Haughey et al (1989) reported no difference.

We have previously reported an increased risk associated with a history of sexually transmitted disease for all testicular tumours (UK Testicular Cancer Study Group, 1994*b*), but considered that reporting bias was a possible explanation for this finding due to the sensitive nature of the question. In a comparison of cases alone, reporting bias should be reduced, and here we found that more cases with non-seminoma tumours than pure seminoma reported a sexually transmitted disease. This finding was consistent for genital herpes, gonorrhoea and other sexually transmitted diseases considered separately and also in younger and older men, although the statistical significance was reduced due to smaller numbers. No other studies to our knowledge have investigated such an effect by histological group.

Our finding of a stronger protective effect of late puberty for non-seminoma than pure seminoma is consistent with two other reports. Moss et al (1986) found a similar difference using a question about age at appearance of pubic hair, as did Møller and Skakkebaek (1996) where boys were asked to compare their age at puberty to that of their classmates. These findings for a range of related questions imply a genuine, though small, difference.

Overall, exercise had a protective effect of similar magnitude in the two histological groups. Participation in specific sporting activities such as contact sports and athletics tended to have a stronger protective effect for seminoma compared with other tumours. As a number of different activities were examined in

 *© 1999 Cancer Research Campaign*

these analyses this may be a chance finding. Other authors have not presented results from similar analyses.

Some authors have explored occupational differences by tumour type. Hayes et al (1990) found an increased risk of non-seminoma for production workers, Knight et al (1996) reported increased risks of non-seminoma for miners and employees in food production, utilities and the leather industry, whereas Haughey et al (1989), like us, found no occupational differences by tumour type. Due to the number of occupations being examined, these analyses again are susceptible to the detection of spurious associations.

None of the differences in risk identified by us and supported by other studies were very large, and would not in their own right be deserving of major consideration. There is, however, increasing evidence that testicular germ cell cancer may arise by clonal evolution with seminoma being seen as an intermediate stage between carcinoma in situ and non-seminoma (Oliver et al, 1995). Cytogenetic studies provide support for this as they demonstrate that the step from seminoma to non-seminoma is associated with loss of chromosomes 12 and 15 (Looijenga et al, 1993). With evidence for an association of declining sperm counts with rising testis cancer rates (Carlsen et al, 1995) and atrophy induced increased gonadotrophin drive as the final common pathway (Oliver, 1990), it is possible to understand how an atrophogenic insult such as testicular trauma could be associated with accelerated progression and chromosome loss. The observation that seminoma was more frequent in the older cryptorchid patients who had a lower incidence of surgical correction would also fit with this observation as uncorrected cryptorchidism would be less traumatic. There is evidence from Swerdlow et al (1997) that the trauma of a biopsy at orchidopexy is associated with an increased risk of tumour.

In conclusion, we have identified, in a large study of testicular tumours, several factors where the risks of pure seminoma and tumours of other histologies differ. We have found patterns of risk, consistent with other published studies which indicate that undescended testis is associated more strongly with pure seminoma tumours than other tumours, and that the protective effect of a late puberty is more marked for non-seminoma tumours. Our finding of differences for testicular trauma, sexually transmitted disease and sporting activities may have arisen by chance, and require confirmation in other studies. Although the differences identified tended to be small, they may indicate factors which act as selection pressures and contribute to the clonal evolution of these tumours.

### Members of the United Kingdom Testicular Cancer Study Group

Study coordinators: K Baker, S Dawson.
Regional collaborators: J Birch, RA Cartwright, PC Elwood, C Tyrell.
Interviewing staff: R Brett, T Bush, V Isbell, A Cornwell, R Steer, S Thistlethwaite, H Gellman, J Hughes, M Llewellyn, A Ardern-Jones, A Allen, E Hilton, B Lloyd, S McVeigh, M Thorne, P Trowbridge, S Reid.

### ACKNOWLEDGEMENTS

This study was funded by Imperial Cancer Research Fund, the Cancer Research Campaign and the Medical Research Council. Further analysis was supported by the Cancer Research Campaign. We wish to thank those who helped with case finding, the family practitioner committees (now FHSAs) who helped with control selection, the consultants and general practitioners who allowed us to interview their patients and the patients and control men who helped with the study.

### REFERENCES

Carlsen E, Giwercman A, Keiding N and Skakkebaek NE (1995) Declining semen quality and increasing incidence of testicular cancer: is there a common cause? *Environ Health Perspect* **103**: 137–139

Coldman AJ, Elwood JM and Gallagher RP (1982) Sports activities and risk of testicular cancer. *Br J Cancer* **46**: 749–756

Coupland CAC, Chilvers CED, Pike MC, Davey G and Forman D (1998) Differences in the aetiology of testicular cancer by histological tumour type. In: *Germ Cell Tumours IV*, Jones WG, Appleyard I, Harnden P and Joffe JK (eds), pp. 9–16. John Libbey: London

Gallagher RP, Huchcroft S, Phillips N, Hill GB, Coldman AJ, Coppin C and Lee T (1995) Physical activity, medical history, and risk of testicular cancer (Alberta and British Columbia, Canada). *Cancer Causes Control* **6**: 398–406

Haughey BP, Graham S, Brasure J, Zielezny M, Sufrin G and Burnett WS (1989) The epidemiology of testicular cancer in upstate New York. *Am J Epidemiol* **130**: 25–36

Hayes RB, Morris Brown L, Pottern LM, Gomez M, Kardaun JW, Hoover RN, O'Connell KJ, Sutzman RE and Jaradpour N (1990) Occupation and risk for testicular cancer: a case-control study. *Int J Epidemiol* **19**: 825–831

Henderson BE, Benton B, Jing J, Yu MC and Pike MC (1979) Risk factors for cancer of the testis in young men. *Int J Cancer* **23**: 598–602

Knight JA, Marrett LD and Weir HK (1996) Occupation and risk of germ cell testicular cancer by histologic type in Ontario. *J Occ Env Med* **38**: 884–890

Looijenga LH, Gillis AJ, Van Putten WL and Oosterhuis JW (1993) In situ numeric analysis of centromeric regions of chromosomes 1, 12 and 15 of seminomas, nonseminomatous germ cell tumours, and carcinoma in situ of human testis. *Lab Invest* **68**: 211–219

Møller H (1993) Clues to the aetiology of testicular germ cell tumours from descriptive epidemiology. *Eur Urol* **23**: 8–13

Møller H and Skakkebæk NE (1996) Risks of testicular cancer and cryptorchidism in relation to socio-economic status and related factors: case-control studies in Denmark. *Int J Cancer* **66**: 287–293

Møller H, Prener A and Skakkebæk NE (1996) Testicular cancer, cryptorchidism, inguinal hernia, testicular atrophy, and genital malformations: case-control studies in Denmark. *Cancer Causes Control* **7**: 264–274

Morrison AS (1976) Cryptorchidism, hernia and cancer of the testis. *J Natl Cancer Inst* **56**: 731–733

Moss AR, Osmond D, Bacchetti P, Torti M and Gurgin V (1986) Hormonal risk factors in testicular cancer. A case-control study. *Am J Epidemiol* **124**: 39–52

Oliver RTD (1990) Atrophy, hormones, genes and viruses in aetiology germ cell tumours. *Cancer Surv* **9**: 263–286

Oliver RTD, Leahy M and Ong J (1995) Combined seminoma/non-seminoma should be considered as intermediate grade germ cell cancer (GCC). *Eur J Cancer* **31**: 1392–1394

Prener A, Engholm G and Jenson OM (1996) Genital anomalies and risk for testicular cancer in Danish men. *Epidemiology* **7**: 14–19

Stone JM, Cruickshank DG, Sandeman TF and Matthews JP (1991) Laterality, maldescent, trauma and other clinical factors in the epidemiology of testis cancer in Victoria, Australia. *Br J Cancer* **64**: 132–138

Strader CH, Weiss NS, Daling JR, Karagas MR and McKnight B (1988) Cryptorchism, orchiopexy, and the risk of testicular cancer. *Am J Epidemiol* **127**: 1013–1018

Swerdlow AJ, Huttly SRA and Smith PG (1987) Testicular cancer and antecedent diseases. *Br J Cancer* **55**: 97–103

Swerdlow AJ, Higgins CD and Pike MC (1997) Risk of testicular cancer in cohort of boys with cryptorchidism. *Br Med J* **314**: 1507–1511

UK Testicular Cancer Study Group (1994*a*) Aetiology of testicular cancer: association with congenital abnormalities, age at puberty, infertility and exercise. *Br Med J* **308**: 1393–1399

UK Testicular Cancer Study Group (1994*b*) Social, behavioural and medical factors in the aetiology of testicular cancer: results from the UK study. *Br J Cancer* **70**: 513–520

*© 1999 Cancer Research Campaign*
